# Insecticide Residues on Poultry Manures: Field Efficacy Test on Selected Insecticides in Managing *Musca Domestica* Population

**DOI:** 10.21315/tlsr2017.28.2.4

**Published:** 2017-07-31

**Authors:** Song-Quan Ong, Abdul Hafiz Ab Majid, Hamdan Ahmad

**Affiliations:** Household & Structural Urban Entomology Laboratory, Vector Control Research Unit, School of Biological Sciences, Universiti Sains Malaysia, 11800 USM Pulau Pinang, Malaysia

**Keywords:** Extraction method, Ultra-Performance Liquid Chromatography (UPLC), poultry manure, *Musca domestica* (L)

## Abstract

In this study, bifenthrin (Maxxthor SC, Ensystex Australasia Pty Ltd), imidacloprid (Prothor SC, Ensystex Australasia Pty Ltd) and fipronil (Regent^®^50SC, Bayer) were applied on the natural infest manures according to the manufacturer rate during a broiler breeding cycle. Solvent direct-immersion extraction (SDIE) was used in detecting the target compound and later, quantification of the insecticide residues in field condition was investigated. The samples were prior cleaned up by solid-phase extraction (SPE) and analysed by Ultra-Performance Liquid Chromatography (UPLC) – photodiode array (PDA) system. In the field trial, three insecticides were showed accumulation during the broiler breeding period and it is suggested that they acted as adulticides when applied on the poultry manures, this is supported by the significant correlation between the increment of insecticide residues to the reduction percentage of adult flies (<0.05). Fipronil showed significantly greater reduction on the adult fly compared to the other insecticides, in which the reduction rate compared to control population at the end of the broiler breeding period; fipronil, imidaclopril and bifenthrin reduced 51.51%, 28.30% and 30.84% of adult flies, respectively.

## Abstract (ru)

Dalam kajian ini, bifenthrin (Maxxthor SC, Ensystex Australasia Pty Ltd), imidacloprid (Prothor SC, Ensystex Australasia Pty Ltd) dan fipronil (Regent^®^50SC, Bayer) telah digunakan pada najis ayam yang dikerumuni oleh lalat *Musca domestica* secara semulajadi dalam satu kitaran penternakan ayam daging. Pengekstrakan rendaman pelarut (SDIE) telah digunakan dalam mengesan kompaun racun dan kemudian, kuantifikasi dan kualifikasi racun serangga pada keadaan bidang telah disiasat. Sampel telah dibersihkan dengan pengekstrakan fasa pepejal (SPE) dan dianalisis oleh Ultra-Performance Liquid Chromatography (UPLC) – sistem fotodiod pelbagai (PDA). Dalam kajian lapangan ayam, tiga racun serangga telah menunjukkan pengumpulan dalam tempoh penternakan ayam dan ia dicadangkan bahawa tiga racun serangga bertindak sebagai racun lalat dewasa walaupun digunakan pada najis ayam, ini telah disokongi dengan korelasi yang signifikan antara kenaikan sisa-sisa racun serangga kepada peratusan pengurangan daripada lalat dewasa (<0.05). Fipronil menunjukkan pengurangan lalat yang lebih tinggi berbanding dengan racun serangga yang lain, di mana kadar pengurangan berbanding untuk mengawal lalat pada akhir tempoh pembiakan ayam daging; fipronil, imidaclopril dan bifenthrin mengurangkan 51.51%, 28.30% dan 30.84% lalat dewasa, masing-masing.

## INTRODUCTION

Insecticides application is a common approach in poultry unit of Malaysia for controlling the population of the major pest, house fly, *Musca domestica* L (Diptera; Muscidae) (Ong *et al.* 2015). Department of Veterinary Services (DVS) Malaysia have reported some of the poultry farms in Malaysia were applied termicides to protect the wooden poultry houses and at the same time, controlling the house fly population. Nevertheless, the efficacy of the termicides toward the house fly is little been studied in Malaysia, although some of them have been used for controlling the adult fly (Diclaro *et al.* 2011, Khan *et al.* 2013). To assess the efficacy and safety of an insecticide, analysis of the insecticide residues on the treated poultry manures is crucial to the efficacy evaluation (Page *et al.* 1988). Yet the effectiveness of the insecticide residues in poultry manures towards the house fly are vague due to the limited reported residues detection methods. Although some standard organisation such as Environment Protection Agency (EPA), America Standard Testing Methods (ASTM) and America Official Analytical Chemists (AOAC) propose some standard methods for study the insecticide residues, but these methods are focusing on soil, water, agricultural products or food (AOAC method 2007, ASTM method E1527, 2013), excluding poultry manures.

Therefore, in this study, the residues of three insecticide; bifenthrin, imidacloprid and fipronil were prior studied using solvent direct-immersion extraction (SDIE) in extracting the residues in poultry manures and subsequently, quantified using Ultra-Performance Liquid Chromatography (UPLC) then compared the efficacy infield. The study aim to study the residues efficacy in controlling house fly on poultry farm Malaysia.

## MATERIALS AND METHODS

### Chemicals

The insecticides that were used in this study was based on the information provided by Department of Veterinary Services-DVS that the insecticides had been used by the poultry farms of Perak, Malaysia in 2013. The insecticides were used as the technical grade products as detailed in Table 1.

For residues study in Ultra-Performance Liquid Chromatography (UPLC), high purity insecticides were used as standard. Bifenthrin (Fluka, 98.5%), fipronil (Fluka, 97.9%) and imidacloprid (Chem Service, 99.5%) were acquired from Sigma-Aldrich Malaysia whereas, the solvents: dichloromethane, acetone and hexane (Fisher Chemical, Malaysia) used for extraction were technical grade. For UPLC analysis, HPLC grade of acetonitrile (Fisher Chemical, Malaysia) and ultrapure water from Milli-Q^®^ purification system were used.

For the field trial, technical grade products Maxxthor SC (Bifenthrin 10%, Ensystex Australasia Pty Ltd), Prothor SC (Imidaclopril 20%, Ensystex Australasia Pty Ltd) and Regent^®^50SC (Fipronil 5.0%, Bayer) were treated on the natural infected poultry manures according to their manufacturer recommended rate.

### Residues Detection

To validate the insecticides detection, five standards ranging in concentrations of 50 to 500 ppm were prepared by weighing the analytical grade of bifenthrin (Fluka, 98.5%), fipronil (Fluka, 97.9%) and imidacloprid (Chem Service, 99.5%) in a 25mL volumetric flask and acetonitrile (HPLC grade, Fisher Chemical, Malaysia) was used as solvent. Later, the solutions were prior filtered by 0.20μm syringe filters (Acrodisc^®^, Pall Corporation) before transferred to the glass vial for UPLC analysis.

### SDIE (Solvent Direct-Immersion Extraction)

Solvent direct-immersion extraction (SDIE) was modified from CDFA (2011) and Hernandez *et al.* (2012). Five grams of sample was weighed and put into a 100 mL beaker with 20 mL of solvent made up of acetone: water: specific solvent in the ratio of 1:1:2. The specific solvent was referred to as high solubility of active ingredient for the respective insecticide based on Toxicology Data Network, TOXNET (2015). Imidacloprid was more soluble in dichloromethane (at 20°C: dichloromethane 67 g/L), bifenthrin was highly soluble in hexane (at 20°C, >600 g/L), and filpronil more soluble in acetone (at 20°C, 545.9 g/L). The samples with the solvent were homogenised using a table shaker at 200 rpm for 2 h and immersed for 24 h. During the immersion, the samples were covered with aluminum sheets and black coloured plastic covers to prevent photo-decay of the compound. After the immersion, the top solvent layer was moved to a clean 10 mL centrifuge tube and subjected to centrifugation using Hettich Universal 320 (Hettich Zentrifugen, UK) at 2000 rpm for 5 min. The centrifugation was repeated twice before the supernatant proceeded to the clean-up and concentrated stages.

### Clean-up and Concentrated Stages

Method EPA 3660c was applied for the clean-up stage. Solid-phase extraction (SPE) C-18 (Supelclean™ ENVI™-18 SPE wt. 500 mg, volume 6 mL) was preconditioned by 4 mL of methanol then 5 mL of ultra-pure water and vacuumed until dry. Five milliliters of supernatant from SDIE were loaded to the SPE, at the rate of 1 drop/s and the filtrate was concentrated until dry at 55 ± 5°C in a ventilated oven. The dried remains were reconstituted in one milliliter of acetonitrile in the amber glass vials for UPLC analysis.

### UPLC (Ultra-performance Liquid Chromatography) Analysis

There are methods that have been employed to measure residues of insecticide prior to this study, such as gas chromatography-mass spectrometry (GC-MS) and high performance liquid chromatography (HPLC) (Obana *et al*. 2003). However, GC is a poor method of doing this due to the weak volatility, polarity and thermal instability of the insecticides. In contrast to GC, LC is more effective and appropriate for the residual analyses of insecticides and in this study, ultra-performance liquid chromatography (UPLC) was applied for compound detection and quantification of the insecticide.

The compounds-imidacloprid, befenthrin and fipronil were analysed using an ACQUITY UPLC^™^ WATER^®^ system consisting of a PU-1580 pump coupled to an HG-1580-31 mixer and an photodiode array (PDA) detector with programmable excitation and emission wavelengths. Separation was achieved using an ACQUITY UPLC^™^ BEH C18 Column (1.7 μm × 2.1 mm × 100mm). The premixed mobile phase used 20% water in methanol at a flow rate of 0.1 mL/min and the injection volume was 5μL. The PDA detector was set with an excitation wavelength of 270 nm for Imidacloprid, 204nm for bifentrin and 280nm for fipronil. The initial mobile phase of water/acetonitrile was 30:70 (v/v) for imidacloprid, 60:40 (v/v) for bifentrin, and 30:70 (v/v) for fipronil (Baskaran *et al.* 1999, Saran *et al.* 2008).

### Calculation

The quantitative measurement for the insecticide’s residue followed that of CDFA (2011):

$$\text{Residues }(\text{ppm})=\frac{\begin{array}{l} \left(\text{Sample peak height area})(\text{Standard concentration},\text{ppm}\right)\\ \left(\text{Standard volume injected},\mu \text{L})(\text{Sample final volume},\text{mL}\right) \end{array}}{\begin{array}{c} \left(\text{Standard peak height area})(\text{Sample volume injected},\mu \text{L}\right)\\ \left(\text{Sample weight},\text{g}\right) \end{array}}$$

### Field Test of the Insecticides and Measurement of Residues

#### Location

The study was conducted from March to June 2014 on a broiler breeder poultry farm in an oil palm plantation in of Ayer Tawar, Perak, Malaysia, at the coordinates 4°21′20.48″N, 100°48′02.59″E. The farm occupies an estimated 10 hectares and contained 16 high-rise wooden poultry houses that measured approximately 60 m long, 10 m wide and 12 m high. Manure accumulated below the houses at depths of 0.5–1.0 m and was removed after the breeding cycle (32–40 days after the introduction of one-day-old chicks).

#### Insecticide residues study

Samples of 300–500 g of manures were taken before each insecticidal treatment for residue study. The study of the insecticide residues were based on the significant sensitive extraction methods, which is the SDIE. Therefore, the samples of manures that were taken before each treatment in the testing period were extracted and cleaned-up as described in section of SDIE and Clean-Up and Concentrated Stages, respectively and detected by UPLC and the quality was calculated as the formula in section Calculation.

#### Insecticides application

The treatment was started on the 10th to 12th day after the introduction of one-day-old broilers due to the removal of heat-retaining cloths and manures were begin to accumulate.

The investigation of quantification and qualification of the insecticides in field was evaluated by applying the insecticides, bifenthrin, imidacloprid and fipronil (Table 1) on the naturally infested manures, which measured in 5 × 8m^2^ at the manures accumulation site under the high-rise broiler poultry houses. Three insecticides were treated on three separated poultry house, respectively and the manufacturer-recommended dose was used for each application. The insecticides were dispersed using a polycarbonate hand sprayer (Mesto^®^ Maxima, Germany). The sprayer was equipped with a 1.10 nm hollow cone nozzle that delivered a cone-shaped spray with low drift and was adjustable to a maximum pressure of 3 bars. The sprayer was also equipped with an 80 cm spray wand and 250 cm spiral hose that delivered the liquid at a rate of 4.5 litres per minute. A pressure indicator permitted the observation of the pressure in the spray tank.

Control for the house fly population usually executed as weekly based interval (WHO, 1986, Williams, 2010) although it might also depends on the insecticides manufacturer recommendation. Nevertheless, the application interval for this study was fixed at 7 days, this is also a common treatment interval for the local poultry owner in controlling the house fly larva (personnel communication).

The control of this study consists of a poultry house that is not treated with any insecticides during the testing period. The treatment houses were aparted to the control house at least 100 m (bifenthrin, imidacloprid and fipronil are aparted 500m, 180m and 800m, respectively to the control house) and oil palm trees that were located between the houses were served as boundaries to prevent major migration of the flies.

Testing plot was defined as a treatment area of 5 × 8 m^2^ of the manure surface where manure accumulation begins. The testing plot consisted of three separated area represented as three replicates. The larval population density in the test plot was defined using infection areas (m^2^), which were measured using a 50 × 50 cm quadrate square with a 10 × 10 cm grid and five quadrates per testing plot were assessed. For adult population density measurements, a scudder grill (Scudder, 1947) measuring 50 × 50 cm was used to sample the adult fly population. This method is the standard for measuring fly populations in many control programs because it neither repels nor attracts flies (WHO, 1983, Bong and Zairi, 2009). Five counts were obtained from the area near the testing plot where flies aggregated.

#### Statistical analysis

For the efficacy of insecticides in field trial, the larval infection area and the adult fly scudder counts of the treatment houses at the end of the breeding period (4th week) were compared to the control population using One–Way Analysis of Variance (ANOVA) in SPSS 17 the significant differences between means were determined by least significant difference (LSD) at p ≤ 0.05.

The residues of the insecticides were direct-proportional to the reduction of adult flies by observation, therefore the correlation between the quantitative (residues) of insecticides and the adult fly population reduction (treatment population compared to the control population) in percentage was studied using Pearson Correlation at α = 0.05. The scudder grill count reduction percentage were prior transformed by arnsin-square root and subjected in SPSS 17.0 Pearson Correlation at α = 0.05.

## RESULTS

**Figure 1:**
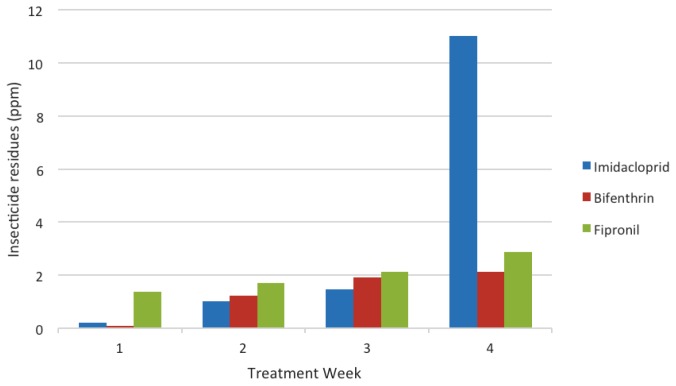
Insecticide residues according to the treatment week

In general, three insecticides demonstrated residual accumulation during the treatment period and the residues at the end of the treatment period showed increment compared to the beginning of treatment period. The residues in the manures for the 4 times treatment is presented in Figure 1, in which imidacloprid, bifenthrin and fipronil increased 39 folds, 0.24 folds and 1.05 folds, respectively at the end of the treatment period.

The effectiveness of the insecticides was represented by the comparison of population between the treatments and control houses at the 4th week of the study. For larvicidal effects of the insecticides, the larval density was represented by their infestation area as suggested by WHO (1986). As can be seen in Table 1, three insecticides demonstrated weak effect on the larva at end of the treatment period (4th week), in which imidaclopril and bifenthrin showed 31.82% and 33.33% increment, respectively while fipronil reduced only 10% of the infestation area. The larval infestation area is detailed in Table 1.

As can be seen in Table 1, Imidacloprid and bifenthrin have a fairly weak reduction percentage for the adult flies during the treatment period. Despite of these, fipronil, which is not a commonly used insecticide in controlling house fly program, showed as much as 51.51% reduction of adult flies compared to the control house. As the residue increase, the reduction percentage increased, therefore, the relationship between the residues and the reduction percentage (transformed by arcsine-square root) for each treatment were studied by Pearson correlation at significant level <0.05. In overall, the residues showed strongly positive correlation with the reduction percentage of adult flies (Table 1).

## DISCUSSIONS

Although previous studies had documented the methods for extracting certain insecticides from semi-solid sludge, but bio-waste such as poultry manure was poorly been studied, even insecticidal application on the manures is common for controlling the house fly larval. By using specific solvent system, SDIE provided more reliable detection limit to measure the insecticide residues in this study. The higher detection limit of SDIE was due to the specific solvent system that based on the solubility of the insecticides, as suggested in King (1980), where a solvent selection in an extraction method should consist of high capacity to the target compound. Imidacloprid and fipronil are considerable polar insecticides (514ppm/L and 1.9mg/L in water, respectively) and therefore, they are more soluble in methanol and acetone, respecively. Bifenthrin, a non-polar compound was more soluble in non-polar solvent such as hexane, isooctane and dichloromethane (PubChem).

Three of the insecticides were found accumulated during the 4 weeks application period and the high persistency could be due to their higher half-life time, which bifenthrin, imidacloprid and filpronil are having 65 days, 38.9 days and 125 days, respectively (EPA 1999, CDPR 2014). Other important factors that would also affect the degradation of the insecticides are microbes activities in the medium and the properties of the insecitides (Singh *et al.*, 2003, Gilani *et al.* 2010). Additionally, bifenthrin was also very low water solubility and high affinity as residue in soils (EPA 1999).

The unsatisfied effect of imidacloprid and bifenthrin on house fly in the present field trial might because of the mechanism and mode of the insecticides. Although imidacloprid was studied by numerous researches but mainly oral poisoning was demonstrated (Kaufman *et al.* 2006, Nurita *et al.* 2008) as a bait (for example QuickBayt^®^) for used against the adult fly. The reasons of inefficiency of bifentrhin in controlling house fly larval might due to the common practice as the target stage for pyrethroid was adult stage (WHO 1986). Additionally, although bifenthrin is declaimed to have a greater photostability (Mokry & Hoagland 1989), but the research of Peck *et al.* (2013) had demonstrated the insecticidal effect of bifenthrin was greatly reduced after exposed to the sunlight for 3 weeks. The inference for the ineffectiveness of bifenthrin could due to the degradation of the compound after exposed to the sunlight. Whereas, fipronil showed minor reduction to the larva population, which reduced 9.09% of larva population compared to the control. Furthermore, the low reduction of imidacloprid and bifenthrin can be due to the dilution of dose because of the accumulation of manures. This is supported by situation of manures accumulation and rich microbes that will enhance the biodegradation process and causing lower residue efficacy (Singh *et al.* 2003, Gilani *et al.* 2010). On the other hand, the slow-acting, fipronil was commonly applied as bait for the control of termite, is firstly studied for used against the house fly in Malaysia, although numerous attempts have had be done for controlling the adult flies (Diclaro *et al.* 2011, Khan *et al*. 2013).

This was suggested the insecticides tested in this study act more likely as adulticides on poultry manures. It is supported as the reduction percentage of adult flies was significantly correlated with the residue of the three insecticides. Even though the imidacloprid and bifenthrin reduced adult fly by 28.30% and 30.84%, respectively compared to the control house, nonetheless, fipronil reduced greatly (51.51%) of fly population compared to the control population.

## CONCLUSIONS

In field trial, as the strong correlations between the residues and adult flies reduction suggested the tested insecticides act more as adulticide when applied on the poultry manures. Fipronil is a potential insecticides for controlling house fly in Malaysia, future attempts should be focused on the application mode (residue or bait).

## Figures and Tables

**Table 1:** Insecticide residues in respond to the larval infestation area and scudder grill (means ± s.e.), and the correlation between the residues and the scudder grill reduction

Active Ingredient (A.I.) and content	Product’s name (Manufacturer) and recommended dose	Week#	Larval infestation area (cm^2^)	Scudder grill	Scudder grill reduction (%)**	Correlation between residues and Scudder grill reduction (α = 0.05, 2-tailed)
Control		1	300 ± 15.62	9.00 ± 0.45		
		2	375 ± 12.11	20.47 ± 0.86		
		3	408 ± 10.02	24.60 ± 1.55		
		4	430 ± 21.89*	28.73 ± 1.61^a^		
Imidacloprid (20%)	Prothor (Ensystex Australasia Pty Ltd)	1	285 ± 11.25	9.18 ± 2.41	−2.01	r = 0.981
	50–100ml/10L	2	350 ± 8.96	18.40 ± 1.08	10.11	Sig = 0.019
		3	490 ± 18.25	21.20 ± 3.70	13.82	
		4	435 ± 16.05*	22.04 ± 4.50^b^	23.30	
Bifenthrin (10%)	Maxxthor (Ensystex Australasia Pty Ltd)	1	255 ± 13.47	8.91 ± 1.54	1.00	r = 0.957
	125–250ml/10L	2	360 ± 6.32	15.52 ± 0.93	24.15	Sig = 0.43
		3	485 ± 17.22	17.43 ± 2.60	29.15	
		4	440 ± 14.08*	19.97 ± 1.23^b^	30.84	
Fipronil (5.0%)	Reagent^®^50SC (Bayer)	1	265 ± 7.56	9.11 ± 0.54	−1.22	r = 0.968
	0.625–75L/ha	2	325 ± 19.55	16.83 ± 0.70	17.78	Sig = 0.32
		3	400 ± 22.11	18.87 ± 0.65	23.29	
		4	400 ± 16.11*	13.93 ± 0.74^c^	51.51	

# The 4th week is defined as the end of the broiler breeding period, the larval and adult population were compared at the end of broiler breeding period.

* n = 5, Not significant at level of 0.05

Different alphabet indicates significant different (p ≤ 0.05), n = 5

** The scudder grill reduction was the comparison of scudder counts between the treatment houses to the control house (no insecticide treatment)

S.E. = Standard Error

ppm = part per million
